# Crystalline-Induced Arthropathy Following Total Knee Replacement

**DOI:** 10.7759/cureus.17619

**Published:** 2021-08-31

**Authors:** Adam M Green, Anthony Gemayel, Eric Silberg

**Affiliations:** 1 Orthopedic Surgery, Beaumont Health, Dearborn, USA; 2 Orthopedic Surgery, Beaumont Health, Dearborn , USA

**Keywords:** knee, painful total knee, total knee replacement (tkr), crystal arthropathy, gout, arthroplasty

## Abstract

Gout rarely occurs in the setting of prior total joint replacement. It can present as an acute or chronic painful joint that may mimic prosthetic joint infection with further similarities found on physical examination and initial workup. Elevated leukocyte count, erythrocyte sedimentation rate, and C-reactive protein are common to both conditions. The confirmatory test to distinguish infection versus inflammatory arthropathy is joint aspiration with crystal or micro-organism identification microscopically. Establishing proper diagnosis is important in guiding appropriate treatment, which may prevent the unnecessary removal of implants. The current study includes a review of the literature and presents a case of bilateral gouty arthropathy after total knee arthroplasty.

## Introduction

The prevalence of gout in the United States doubled between the 1960s and 1990s and since has quadrupled, affecting approximately 8 million people nationwide [1-3]. Similarly, the incidence of total knee arthroplasties performed annually in the United States is on the rise. To our knowledge, there is limited data on the concurrence of gouty arthritis and primary total knee arthroplasty [4-6].

Acute gouty arthritis is typically the result of intra-articular synovial and cartilaginous crystalline deposition secondary to deranged purine metabolism, more commonly affecting middle-aged males [3]. While the involvement of a single joint is common, polyarticular involvement is more common in men with hypertension and alcohol abuse [2]. The prevalence of gout is on the rise and knowledge of distinguishing features from other acute arthropathies, especially septic arthritis, is important given the different treatment options. In the native joint, aspiration is the only definitive diagnostic procedure, however, in the setting of joint arthroplasty, there is a clinical diagnostic challenge whether to aspirate or not. We present a case of acute bilateral prosthetic knee inflammatory arthritis to increase the awareness of this rare condition, to highlight the distinguishing characteristics from prosthetic joint infection, and to discuss treatment options.

## Case presentation

A 71-year-old man presented to the emergency department complaining of right knee pain and swelling for 24 hours without antecedent trauma. He described 10/10 diffuse lateral knee pain that was non-radiating and not associated with any particular knee position. He denied left knee pain. He denied any recent increase in alcohol or red meat consumption. Past medical history was significant for diabetes mellitus, hypertension on diuretics, and hyperlipidemia.

He was tachycardic with a heart rate of 109 to 123 and was afebrile and normotensive. Laboratory values were notable for a serum white blood cell count of 12.9 bil/L (normal 3.5-10.1) with a left-shift (82.7% neutrophils), erythrocyte sedimentation rate of 73 mm/hr (normal 0-20mm/hr), and C-reactive protein of 218.5 mg/L (normal < 9.0). Blood cultures were taken and the orthopedic service was consulted for further evaluation.

**Figure 1:**
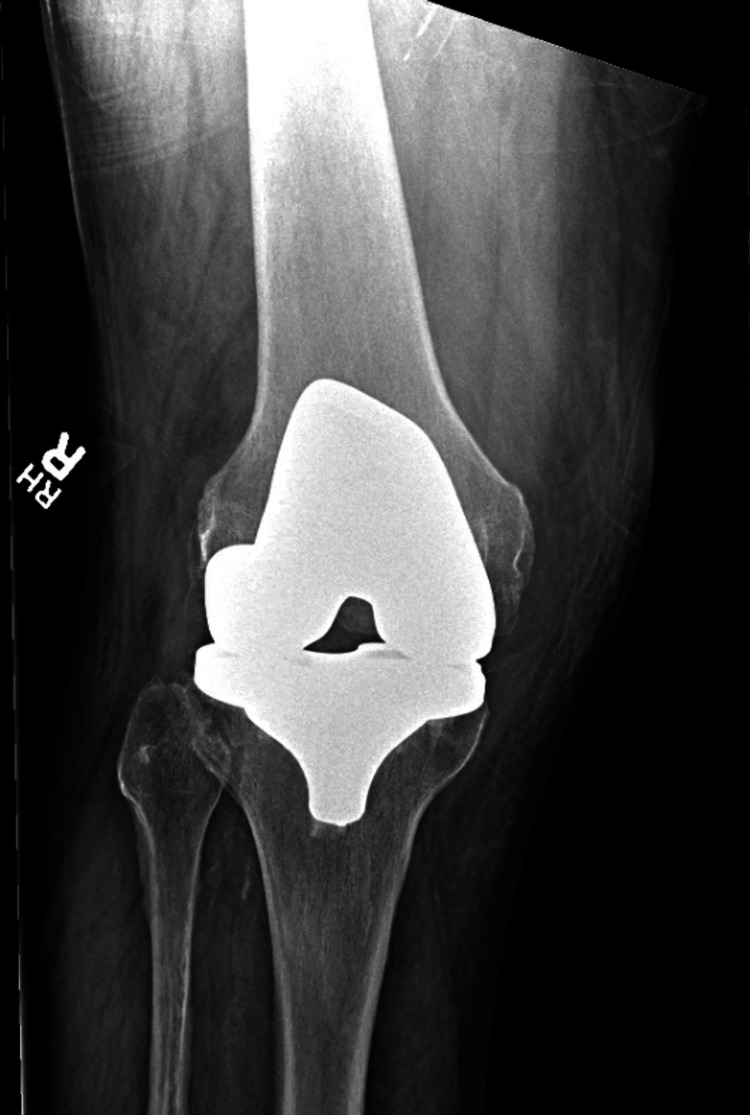
Anteroposterior radiograph of the right knee in a patient presenting with acute crystal-induced arthritis.

**Figure 2:**
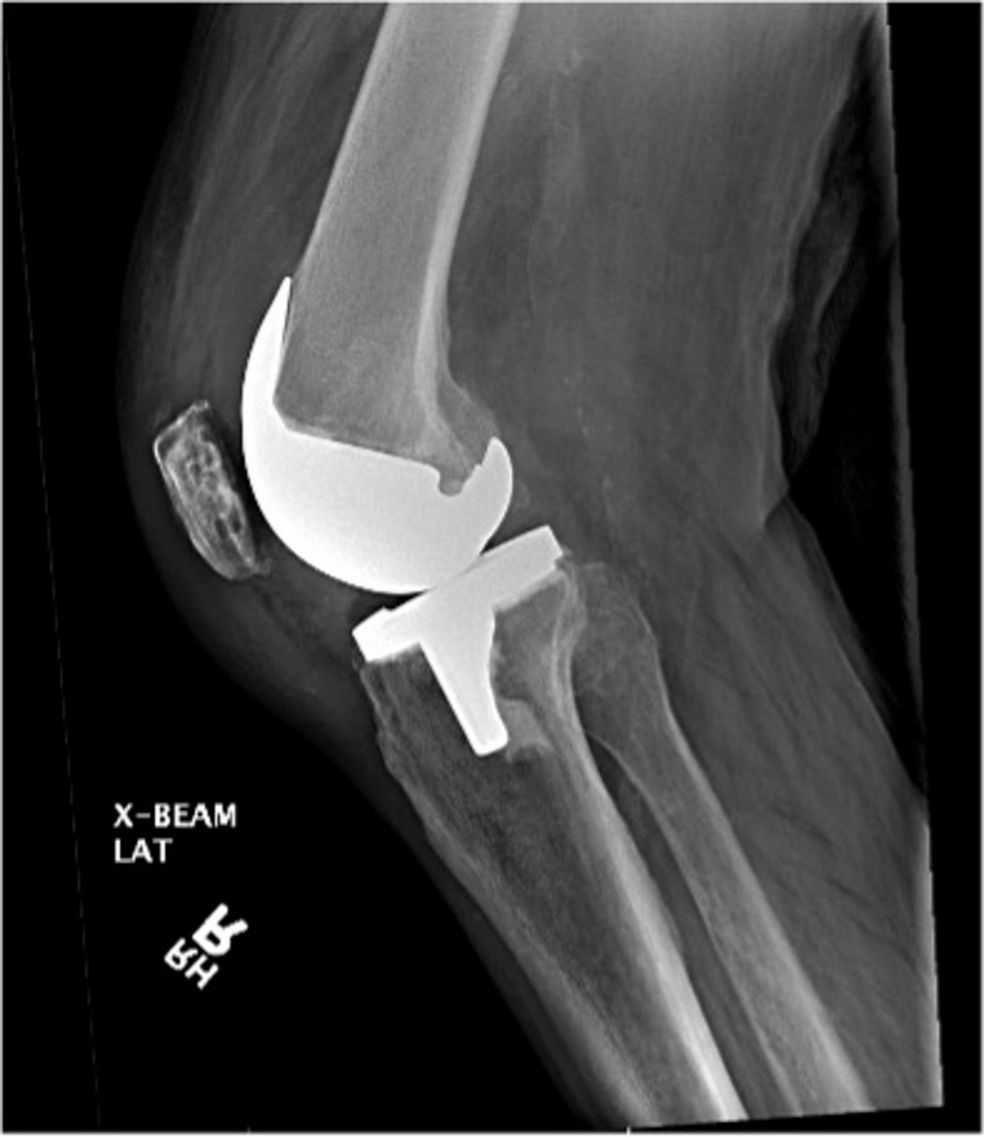
Lateral radiograph of the right knee in a patient presenting with acute crystal-induced arthritis.

Upon evaluation in the emergency department, he described excruciating right knee pain with any movement and inability to bear weight on the right leg. He stated that he had a posterior-stabilized left total knee arthroplasty 12 years prior and a posterior-stabilized right total knee arthroplasty 11 years prior by the same orthopedic surgeon. On examination, the right knee was diffusely tender on palpation, moderately swollen, and warm. He has a prior midline surgical incision that was well-healed, and his knee was not erythematous. Active range of motion was limited from 5-60 degrees of flexion secondary to pain and motor strength was 4/5 in knee extension. Left knee exam showed no effusion, warmth, or erythema, full, painless range of motion, and 5/5 motor strength in flexion and extension. Radiographs of the right knee were taken and revealed a suprapatellar joint effusion, a periosteal reaction involving the proximal lateral tibia metaphysis, and well-aligned surgical components without evidence of loosening or osteolysis, as seen in Figures 1, 2.

The patient subsequently underwent synovial fluid aspiration, was admitted to the internal medicine service with an infectious disease on consult, and started on intravenous antibiotics. Blood cultures were pending. Initial synovial fluid from the right knee showed mucoid straw-colored fluid with 72,320 per cubic mm white blood cells, 99% neutrophils, 11,520 per cubic mm red blood cells, and no organisms. Based on these findings, prosthetic joint infection was the leading diagnosis and the patient was boarded for surgery.

The patient was taken for right knee irrigation and debridement and poly exchange, left knee aspiration, and possible left knee irrigation and debridement and poly exchange the following day. Intra-operative findings included brownish synovial fluid with thickened, inflamed synovium, minimal polyethylene wear, and stable components of the right knee. The left knee was aspirated and revealed a brownish-turbid synovial fluid and the decision was made to proceed with irrigation and debridement. Intra-operative findings again included a thickened, inflamed synovium, intact components with polyethylene wear. Bilateral polyethylene liner exchanges were performed. Bilateral knee irrigation with antibiotic solution and betadine was also performed. Synovial tissue from bilateral knees was sent to pathology and cultures were taken.

**Figure 3:**
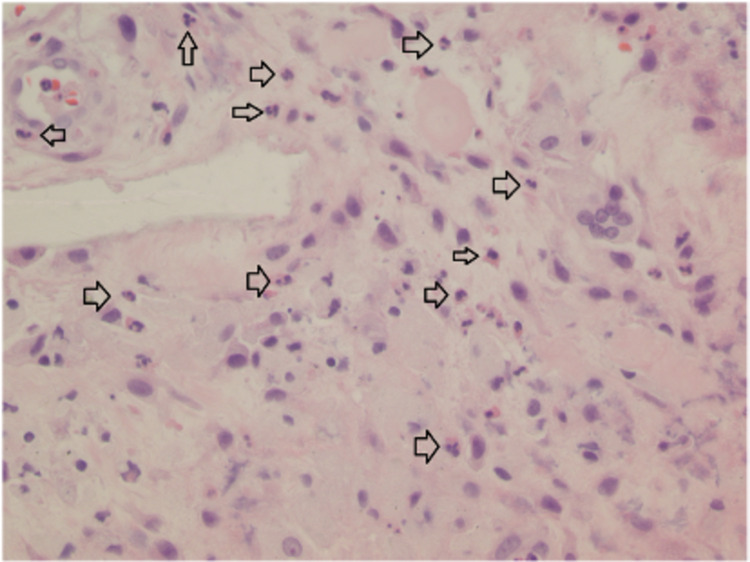
High-power magnification photomicrograph showing inflamed synovium of the right knee and polymorphonuclear cell infiltrate (arrows).

**Figure 4:**
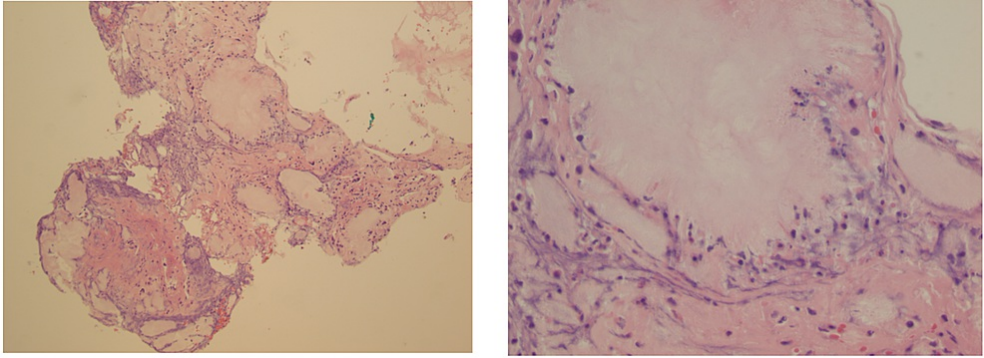
Low and high power magnification of synovial biopsy of the right knee in showing gouty tophi.

Later, synovial fluid results from the initial aspiration were resulted and showed positive monosodium urate crystals. Serum uric acid on post-operative day two was 10.6 mg/dL (3.5-7.2). Final bacterial and fungal blood cultures were negative. Intra-operative cultures showed many neutrophils but no aerobic, anaerobic, or fungal growth. Surgical pathology results are shown in Figure 3. Histology of bilateral knee tissue showed inflamed synovium with increased neutrophils and crystal deposition within the synovium as seen in Figures 3, 4. Based on these findings a diagnosis of bilateral prosthetic joint gouty arthritis was made. The patient was successfully treated with irrigation and debridement, synovectomy, and polyethylene liner exchange. Post-operatively, the patient was started on prophylactic allopurinol to prevent a recurrence.

The patient was subsequently seen and evaluated at routine post-operative follow-up appointments initially at two weeks from the date of surgery and monthly intervals thereafter. The patient began outpatient physical therapy six weeks from the date of surgery. At the two-month follow-up visit following the completion of physical therapy, the patient was noted to have a mild effusion and pain in the left knee. Radiographs taken in the office were unremarkable. The range of motion of bilateral knees was 0-100 degrees. The left knee was aspirated in the office and fluid analysis showed few polymorphonuclear cells and no growth of organisms. The patient was continued on gout prophylaxis medications. At the most recent follow-up visit, the patient was ambulating without difficulty, had full range of motion, and the incisions were healed.

## Discussion

Acute gouty arthritis is a condition consisting of crystal deposition within the synovium and cartilaginous structures of a joint. Symptoms of crystal deposition should improve the following arthroplasty, however, there have been limited reports in the orthopedic literature of crystal-induced arthropathy in the setting of joint prosthesis [4-9]. Furthermore, there is no consensus within the current literature on the optimal treatment of this pathology.

Chen et al. report two cases of acute aseptic gouty arthritis of the knee in patients with a history of gout [4]. Both patients presented with the classic signs and symptoms of an acute gouty flare, underwent joint arthrocentesis to confirm gout, and were managed conservatively with a short course of oral steroids and anti-gout medications. Salin et al. also report on two patients with prior total knee arthroplasty who developed post-operative gouty arthritis [5]. One patient was treated with irrigation and debridement with polyethylene liner exchange, who went on to develop gouty arthritis in the contralateral knee four weeks later which was managed conservatively. The other patient presented with a painful, swollen, erythematous knee with a history of joint replacement and was found to have a concomitant prosthetic joint infection, which was managed with irrigation and debridement with polyethylene liner exchange. Archibeck et al. report a case of tophaceous gout leading to aseptic loosening of a prior knee arthroplasty that was treated with staged revision [6]. At the time of operation, the authors noted chalky material throughout the synovium and deposits within the metaphyseal bone surrounding the components [6].

Distinguishing inflammatory from infectious arthropathy in the setting of prior arthroplasty presents a clinical dilemma for the orthopedic surgeon determining the most appropriate treatment options, which vary from non-operative management to multi-stage revision arthroplasty as reported in the current literature. There have been previous reports of crystal-induced arthropathy treated conservatively or surgically.

There is a remarkable overlap in the symptoms induced by intra-articular crystal deposition and infection following total knee arthroplasty. The most recent diagnostic criteria for prosthetic joint infection, a modification of the Musculoskeletal Infection Society definition of periprosthetic joint infection (PJI) in 2013, includes a scoring system of pre-operative and intra-operative findings, in which infection is diagnosed when one major criterion is present or the total score for minor criteria is greater than six [10]. This evidence-based and validated scoring system has been shown to have an overall sensitivity and specificity of 97.7% and 99.5%, respectively [10]. In our case, the patient presented with a pre-operative score of eight, and although no major criteria were met, a diagnosis of prosthetic joint infection was met based on these findings. Thus, there is no way to distinguish prosthetic joint infection from gouty arthropathy based on these criteria. In our case, intraoperative cultures failed to grow any microorganisms, but tissue specimens showed crystal deposition within the synovium, which further supported the diagnosis of gouty arthropathy.

Recent literature has focused on the identification of infectious markers such as synovial alpha-defensin to aid in the diagnosis of prosthetic joint infection [11-13]. Alpha-defensin belongs to a group of secretory peptides released by neutrophils in response to pathogens [14]. Many synovial fluid assays have since been developed to rapidly identify the presence of alpha defensin in a given sample, with final results obtained in less than 15 minutes [11]. Furthermore, when the alpha-defensins measurement was combined with the serum C-reactive protein level, the sensitivity and specificity of diagnosing prosthetic joint infection were 97% and 100%, respectively [12]. Currently, there are no reports or studies that include synovial fluid alpha-defensin levels in crystal-induced arthropathy. This may be an area for future investigation to distinguish between prosthetic joint infection and crystal-induced arthropathy.

## Conclusions

Gout rarely occurs in the setting of total joint arthroplasty. The mechanism by which this occurs may be related to crystal deposition within the surrounding synovial tissue, invoking the inflammatory cascade. It can present as an acute or chronic painful joint that may mimic prosthetic joint infection, with further similarities found on physical examination and initial workup. Establishing the proper diagnosis is important in guiding appropriate treatment, which may prevent the unnecessary removal of arthroplasty components.
